# Early Improvement Predicts Clinical Outcomes Similarly in 10 Hz rTMS and iTBS Therapy for Depression

**DOI:** 10.3389/fpsyt.2022.863225

**Published:** 2022-05-11

**Authors:** Nathen A. Spitz, Benjamin D. Pace, Patrick Ten Eyck, Nicholas T. Trapp

**Affiliations:** ^1^Department of Psychiatry, University of Iowa, Iowa City, IA, United States; ^2^Institute for Clinical and Translational Science, University of Iowa, Iowa City, IA, United States; ^3^Iowa Neuroscience Institute, University of Iowa, Iowa City, IA, United States

**Keywords:** depression, transcranial magnetic stimulation, theta-burst, clinical practice, observational study, prediction

## Abstract

**Background:**

Prior studies have demonstrated that early treatment response with transcranial magnetic stimulation (TMS) can predict overall response, yet none have directly compared that predictive capacity between intermittent theta-burst stimulation (iTBS) and 10 Hz repetitive transcranial magnetic stimulation (rTMS) for depression. Our study sought to test the hypothesis that early clinical improvement could predict ultimate treatment response in both iTBS and 10 Hz rTMS patient groups and that there would not be significant differences between the modalities.

**Methods:**

We retrospectively evaluated response to treatment in 105 participants with depression that received 10 Hz rTMS (*n* = 68) and iTBS (*n* = 37) to the dorsolateral prefrontal cortex (DLPFC). Percent changes from baseline to treatment 10 (t_10_), and to final treatment (t_f_), were used to calculate confusion matrices including negative predictive value (NPV). Treatment non-response was defined as <50% reduction in PHQ-9 scores according to literature, and population, data-driven non-response was defined as <40% for 10 Hz and <45% for iTBS.

**Results:**

For both modalities, the NPV related to degree of improvement at t_10_. NPV for 10 Hz was 74%, 82% and 73% at t_10_ in those who failed to improve >20, >10, and >0% respectively; while iTBS NPV rates were 65, 71, and 60%. There were not significant differences between protocols at any t_10_ cut-off assessed, whether research defined 50% improvement as response or data driven kernel density estimates (*p* = 0.46–0.79).

**Conclusion:**

Patients who fail to achieve >10% improvement by t_10_ with both 10 Hz rTMS and iTBS therapies have 70–80% chance of non-response to treatment. With no significant differences between predictive capacities, identifying patients at-risk for non-response affords psychiatrists greater opportunity to adapt treatment strategies.

## Introduction

Major Depressive Disorder (MDD) is a ubiquitous mental health disorder that affects a diverse population across the globe and responds to treatment in a seemingly unpredictable manner. Repetitive transcranial magnetic stimulation (rTMS) exists as an increasingly researched, non-invasive treatment for people with MDD (1). Notwithstanding its demonstrated clinical efficacy, treatment responses are variable and difficult to predict (2–5). A full 4 to 6-week treatment course is a time and resource-intensive process which can be especially burdensome, especially for the 30–40% of patients destined for non-response (6).

Literature has defined several biomarkers that may help clinicians predict a patient's response to TMS treatment (7, 8); however, the collection and analysis of these markers is often expensive, inaccessible, or time-consuming for patients and providers. Reliable predictors would thus be of immense clinical utility by prioritizing TMS for subjects most likely to respond to optimize clinical outcomes and to potentially avoid ineffective therapies. To address the inaccessibility of biomarker collection and utilization, a meta-analysis of 41 different pharmacotherapy clinical trials demonstrated that early treatment improvement, defined as >20% symptom reduction in the first 2 weeks of treatment, was able to accurately predict treatment response and remission (9).

A seminal study by Feffer et al. adapted analyses of clinical response to treatment at 2 weeks, previously only done for pharmacotherapy or electroconvulsive therapy (10) to rTMS, in order to determine the accuracy of early clinical response in predicting subsequent response to treatment via rTMS (11). In a naturalistic retrospective case series (*N* = 101), they defined distinct subgroups of responders and non-responders based on standard criteria, as well as on population specific data-driven response criteria using kernel density estimates. The study determined that the absence of early clinical improvement by treatment 10 during a course of right sided dorsomedial prefrontal cortex (DMPFC) 10 Hz rTMS or iTBS (intermittent theta burst stimulation) carried a negative predictive value (NPV) of 88% (11).

Subsequent studies examined other potential predictors of treatment response: one demonstrating a NPV of 72.3% when participants had <20% improvement at week two while using final outcomes of extended treatment courses of 10 Hz stimulation at the left dorsolateral prefrontal cortex (DLPFC) (12), and another finding a NPV of roughly 80% for a population receiving 1 Hz rTMS (13). Calculating metrics such as negative predictive value of early treatment response in clinical TMS populations allows clinicians to better prognosticate who will respond to subsequent therapy and aids in the decision making regarding altering or adapting treatment plans to optimize outcomes. As TMS research explores various stimulation frequencies, durations, targets, and targeting methods in the treatment of major depressive disorder, it is imperative to examine the comparative effectiveness of these varying parameters.

Since being cleared by the FDA in 2008, the recognized standard of care for TMS treatments for MDD has been 10 Hz rTMS to the left DLPFC, which delivers 3,000 pulses in over 37.5 min (14). Recently, a study by Blumberger et al. demonstrated that intermittent theta burst stimulation (iTBS), which delivers 600 pulses in just over 3 min, was non-inferior to 10 Hz rTMS in treating major depressive disorder (15), garnering FDA clearance in 2018 for the treatment of MDD. Few studies exist that directly compare these two modalities in their effectiveness at treating depression, and to our knowledge, no studies have examined if any differences exist between 10 Hz rTMS and iTBS in the use of early treatment improvement to predict treatment response.

Taking this into account, in our single-site, naturalistic observation study, we detail the results of a retrospective chart review that used a similar approach to the aforementioned studies to determine the accuracy of predicting final outcomes based on early treatment response in 10 Hz rTMS and iTBS. We also explore if potential differences exist in the predictive capacities between the two modalities. Predicated on prior research, we hypothesized a criterion of at least 20% improvement by treatment 10 would provide the highest negative predictive value for non-response to a full treatment course, as well as hypothesizing that there would not be significant differences between 10 Hz rTMS and iTBS across various improvement criteria.

## Materials and Methods

### Patient Population

**Figure 1:**
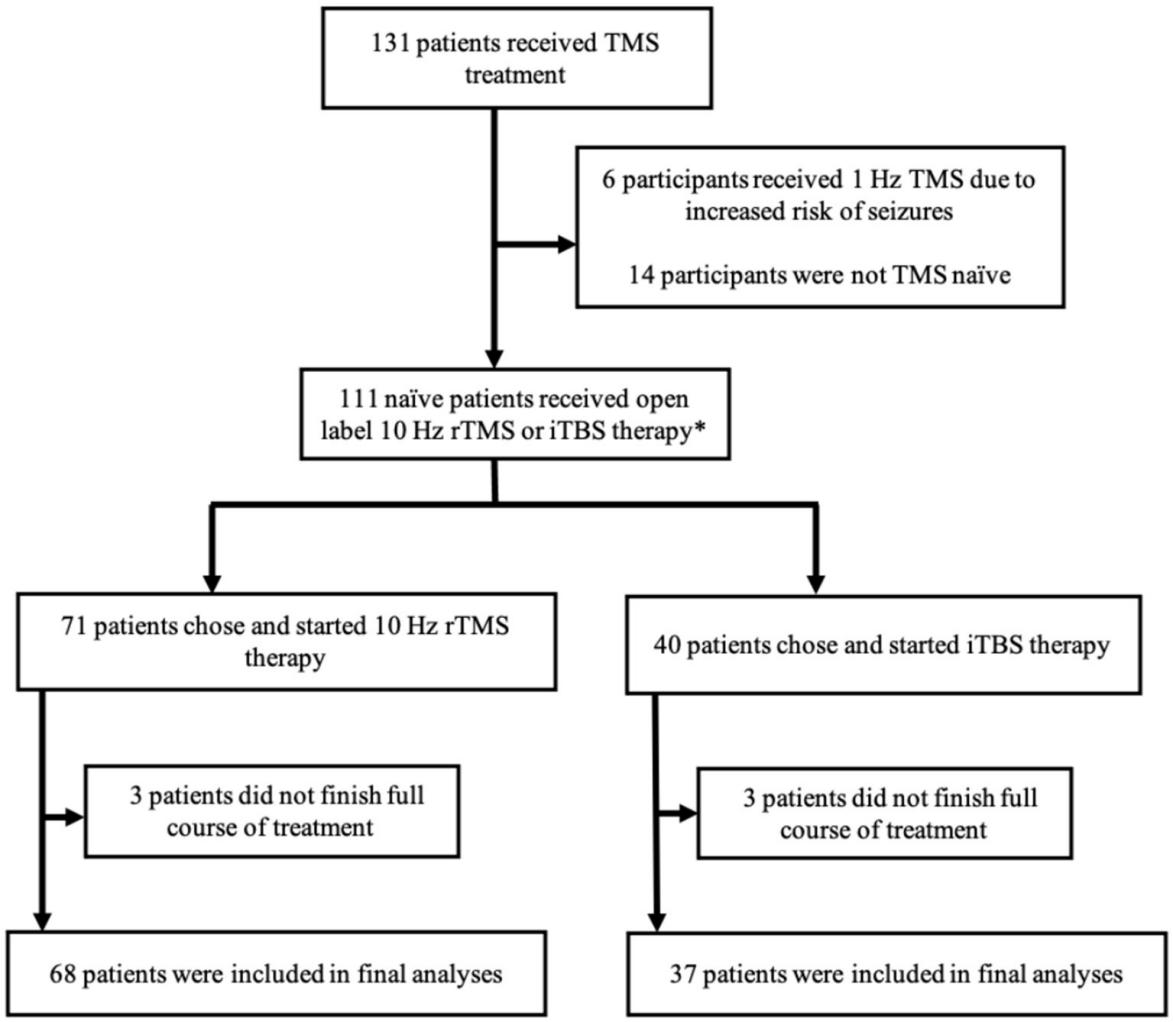
Summary of study participant disposition. A graphical depiction of the various outcomes and participation status of the study participants. TMS, transcranial magnetic stimulation; rTMS, repetitive transcranial magnetic stimulation; iTBS, intermittent theta burst stimulation. *Prior to FDA clearance of iTBS in 2018, participants mainly received 10 Hz rTMS. After the FDA clearance, participants were able to choose between 10 Hz rTMS and iTBS therapy.

This study was conducted using a retrospective chart review of 131 participants that received standard clinical treatment of left DLPFC 10 Hz rTMS or iTBS between December 2016 and February 2020. Inclusion criteria in this study required patients (age ≥ 18) to have an existing diagnosis of MDD. Participants in the study were subsequently evaluated by a physician with experience in TMS and were recommended as suitable candidates to receive TMS treatment based on a thorough diagnostic history and physical, medication reconciliation, assessment of other DSM-5 mental health disorders, and review of previous therapy trials. Exclusion criteria included age <18 years old, a prior diagnosis of epilepsy or other seizure disorders, implanted ferromagnetic hardware in the face or skull near TMS targeting sites, or previous treatment with TMS of any kind. Patient consent was obtained prior to treatment. This study was approved the Institutional Review Board at the University of Iowa. Figure 1 depicts the array of outcomes of the 131 participants who received TMS treatment during the previously described timeframe. We included 105 participants in our final analyses.

### DLPFC-rTMS and iTBS Technique

From 2016 until iTBS was cleared by the FDA for its indication in treating major depressive disorder in 2018, patients in our study received 10 Hz rTMS. After iTBS approval, the prescribing physician and the participants decided on 10 Hz rTMS vs. iTBS therapy through shared decision-making. Resting motor threshold (RMT) was determined via right-handed thumb twitches in three of five trials while delivering stimulation to the left primary motor cortex via the Magventure MagPro X100 Figure 8 Butterfly Coil with Active Cooling (Magventure, Alpharetta, GA) (16). Technicians trained in TMS delivery then targeted the left DLPFC using either the 5.5 cm rule, or the Beam F3 techniques (16–18). Participants receiving 10 Hz stimulation received 3,000 pulses with 4 s trains and a 26-s intertrain interval at 120% of their RMT over a 37.5-min session (14). This contrasts with patients receiving iTBS that received 600 pulses with 50 Hz triplets patterned into 5 Hz stimulation with 2 s trains and 8 s intertrain intervals, also at 120% the intensity of their RMT in a 3-min treatment session (19, 20). In this study, participants received a varying number of sessions over their treatment course (average of 33) following clinical indication, with stimulation sessions occurring for five consecutive days a week for four to six subsequent weeks.

### Clinical Assessments

Every participant in this study completed a baseline clinical assessment via a self-report scale [Patient Health Questionnaire 9 (PHQ-9)] prior to the start of treatment (21, 22). Participants subsequently completed the PHQ-9 at the start of their treatment course (t_1_), at the end of each treatment week, treatment 10 (t_10_), and at the final treatment session (t_f_) to track depression symptomatology and improvement over time. The percent changes in PHQ-9 scores at t_10_ and t_f_ were subsequently used to determine outcome measurements such as negative predictive value to ascertain if early improvement scores could be used to predict future treatment response, as well as if discrepancies between this predictive capacity existed between the two treatment modalities. Secondary outcome measures included using the PHQ-9 t_f_ percent reductions within kernel density estimates to determine the distribution of response levels, allowing classification of distinct data-driven subgroups of “non-responders” and “responders” for analysis that possibly varied from the classically defined >50% reduction dichotomy to define treatment response.

### Data-Analysis

Therapy-stratified summary statistics for continuous and categorical measures are represented as means (standard deviations) and counts (percentages), respectively. Tests for differences in measures between therapies utilized Student's *t*-test and Pearson's chi-square test. Using IBM SPSS Statistics (Version 26), we used cutoff criteria of TMS non-response with 0, 10, and 20% improvement thresholds at t_10_ to populate confusion matrices that detailed sensitivity, specificity, positive predictive value (PPV), negative predictive value (NPV) and total accuracy of t_f_ outcomes. Similar to previous studies that analyzed early treatment response and its predictive capacities in rTMS (11–13), these t_f_ outcomes were subsequently used to define our patient population as responders or non-responders two ways: first using the classically defined criterion of >50% improvement by the final treatment, and secondly, using kernel density estimates (KDE) with an Epanechnikov kernel.

The KDEs allowed us to use a data-driven approach to determine if there were distinct subgroups of “non-responders” based on our data population. This was considered an important analysis based on prior research demonstrating that patient populations do not respond homogenously, and a data-driven cutoff may better dichotomize populations phenomenologically rather than an arbitrary 50% cutoff. This resulted in the use of more liberal response criteria for both the 10 Hz and iTBS groups, respectively. To directly compare if significant differences of predictive capacity existed between 10 Hz rTMS and iTBS treatment modalities, we measured the NPV across the various improvement thresholds at t_10_. Comparisons were made using two-sample proportional *z*-tests to examine if significant differences existed between the two modalities across the both the classically defined >50% improvement criterion for a response or the KDE data-driven response criterion. A *p*-value < 0.05 was considered statistically significant.

## Results

### Demographics

Of the 105 participants included in the final analyses who received standard clinical left DLPFC stimulation, 68 received 10 Hz rTMS and 37 received iTBS between 2016 and 2020. Table 1 depicts the baseline demographics of the participant population. They were 58.5% female, mean (SD) age of 52.3 ± 16.3. At baseline, the only significant difference between treatment groups was comorbid post-traumatic stress disorder with 13 (19.1%) participants in the 10 Hz group and five (13.5%) participants in the iTBS group, *p* = 0.019 (Table 1). No other differences between the two modalities were found in variables analyzed, including age, sex, baseline PHQ-9 score, use of benzodiazepines, or use of stimulant medications.

**Table 1:** Baseline demographic and clinical characteristics of study participants (105).

	**10 Hz rTMS (*n* = 68)**	**iTBS (*n* = 37)**	***p*-value**
Age	53.47 ± 15.7	49.62 ± 17.3	0.251
Women	41 (60.0%)	21 (57.0%)	0.728
Baseline PHQ-9 (range 0–27)	17.8 (4.9)	19.0 (4.4)	0.270
Generalized anxiety disorder	46 (67.7%)	16 (43.2%)	0.178
Post-traumatic stress disorder	13 (19.1%)	5 (13.5%)	0.019*
Benzodiazepines	45 (66.1%)	13 (35.0%)	0.161
Stimulants	14 (20.6%)	11 (29.7%)	0.928

*Data in the table are means (SD) or the number of participants in with group (% total). Statistical significance of between-group analyses was assessed with Student's t-test for continuous data and Pearson's chi-square test for categorical data*.

*p < 0.05.

### Outcomes

Previously reported findings demonstrated that using our dataset there were no statistically significant differences between 10 Hz rTMS and iTBS groups regarding response rates, remission rates, or minimum clinically important difference (MCID) rates (23).

### Categorization of Responders and Non-responders

**Figure 2:**
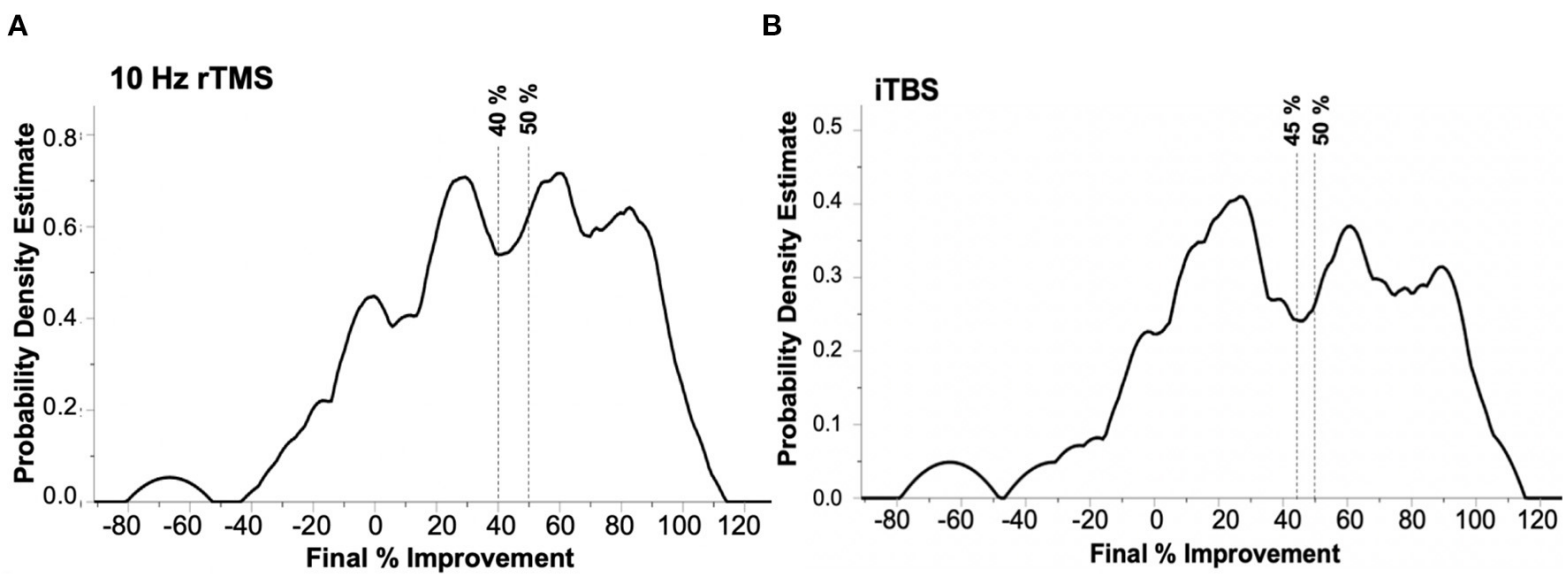
Kernel density estimate (KDE) depicting the modality specific distribution of treatment outcomes as determined by percentage improvement of PHQ-9 scores from baseline to final treatment. **(A)** Kernel density estimates (KDE) with Epanichnikov kernels of participants that received 10 Hz rTMS (*n* = 68) demonstrating a non-normal distribution with distinct sub-group of “non-responders” at 40% compared to the traditional 50% final improvement cut-off. **(B)** KDE of participants receiving iTBS (*n* = 37) with distinct “non-responder” sub-group at 45% compared to traditional 50% final improvement cut-off.

Within the kernel density estimates, similarly to prior studies' methodology (11–13), we used the first major troughs as the cut-off for the unique “non-responder” subgroup. The distribution of participants in the 10 Hz group was trimodal (Figure 2A) with a discrete non-responder group of individuals achieving < 40% improvement, and the distribution in the iTBS group was trimodal as well, with a distinct non-responder group achieving < 45% improvement (Figure 2B). This allowed us to create a data-driven, t_f_ response criterion in both the 10 Hz and iTBS groups using these 40 and 45% improvement cut-offs, respectively. Results from the confusion matrices were compared to those achieved with the standard non-response criterion of <50% improvement as a secondary outcome for completeness.

### Negative Predictive Value Analyses

Using the classically defined >50% response criterion for response, we first determined the NPV at three t_10_ cut-offs (0, 10, and 20%) of improvement at t_10_ for 10 Hz and iTBS using confusion matrices, and next used proportional z-tests to determine if there was a significant difference between the two modalities as detailed in Table 2. For participants who failed to reach >20% improvement at t_10_, the NPVs for 10 Hz rTMS and iTBS were 73.7 and 65.0%, respectively: *p* = 0.49. When the improvement criterion was decreased to >10% improvement the NPVs for 10 Hz and iTBS were 81.5 and 71.4%: *p* = 0.46. Lastly, at >0% improvement the NPVs for 10 Hz and iTBS decreased to 73.3 and 60.0%: *p* = 0.58.

**Table 2:** Early improvement confusion matrices determining final treatment predictive capacity differences between 10 Hz rTMS and iTBS.

	**10 Hz rTMS (*n* = 68)**	**iTBS (*n* = 37)**	***p*-value**
**Classically defined** **>** **50% improvement**			
**>20% improvement by treatment 10**			
Sensitivity	69.7	58.8	0.44
Specificity	80.0	65.0	0.22
PPV	76.7	58.8	0.20
NPV	73.7	65.0	0.49
Total accuracy	75.0	62.2	0.17
**>10% improvement by treatment 10**			
Sensitivity	84.8	76.5	0.47
Specificity	62.9	50.0	0.35
PPV	68.3	56.5	0.35
NPV	81.5	71.4	0.46
Total accuracy	73.5	62.2	0.23
**>0% improvement by treatment 10**			
Sensitivity	87.9	88.2	0.98
Specificity	31.4	15.0	0.18
PPV	54.7	46.9	0.48
NPV	73.3	60.0	0.58
Total accuracy	58.8	48.6	0.32
**KDE defined improvement (>40% 10 HZ**, **>45% ITBS)**			
**>20% improvement by treatment 10**			
Sensitivity	67.6	61.1	0.64
Specificity	83.9	68.4	0.20
PPV	83.3	64.7	0.15
NPV	68.4	65.0	0.79
Total accuracy	75.0	64.9	0.27
**>10% improvement by treatment 10**			
Sensitivity	83.8	77.8	0.59
Specificity	67.7	52.6	0.29
PPV	75.6	60.9	0.22
NPV	77.8	71.4	0.65
Total accuracy	76.5	64.9	0.20
**>0% improvement by treatment 10**			
Sensitivity	86.5	88.9	0.80
Specificity	32.3	15.8	0.20
PPV	60.4	50.0	0.35
NPV	66.7	60.0	0.79
Total accuracy	61.8	51.4	0.30

*Using PHQ-9 score percent changes at treatment 10 and the final treatment, confusion matrices were calculated for 10 Hz rTMS and iTBS across an array of improvement criteria. Classically defined improvement in scores is >50% from baseline. Kernel density estimate calculations were used to determine data-driven non-responder populations to create more stringent and improvement criteria, which was determined to be >40% for 10 Hz rTMS and >45% for iTBS. rTMS, repetitive transcranial magnetic stimulation; iTBS, intermittent theta burst stimulation; PPV, positive predictive value; NPV, negative predictive value; KDE, kernel density estimate*.

Subsequently, using the KDE data-driven, population defined criteria for response for 10 Hz rTMS at >40 and >45% iTBS, using the same parameters, we determined the NPV at three cut-offs (0, 10, and 20%) of improvement at t_10_ for 10 Hz and iTBS using confusion matrices, and subsequently used proportional *z*-tests to determine if there was a significant difference between the two modalities. At >20% improvement at t_10_, the NPVs for 10 Hz rTMS and iTBS were 68.4 and 65.0%, respectively: *p* = 0.79. Then at >10% improvement the NPVs for 10 Hz and iTBS were 77.8 and 71.4%: *p* = 0.65. Lastly, at >0% improvement the NPVs for 10 Hz and iTBS decreased to 66.7 and 60.0%: *p* = 0.79.

## Discussion

The results from our naturalistic observational study suggest that early improvement can be useful for prognosticating who will respond to treatment and suggest similar patterns exist for both 10 Hz rTMS and iTBS targeting the left DLPFC. These findings held true when comparing the two modalities across an array of early improvement criteria (0, 10, and 20%) at treatment 10, and they were unaffected by choice of conventional (>50%) vs. data-driven (>40–45% by kernel density estimates) metrics of response categorization. Our data demonstrated no significant differences between the two modalities.

Moreover, despite no identified significant differences, it is evident that 10 Hz rTMS stimulation had a clear trend of higher NPVs and was more reliable at predicting response at each improvement criterion, as well as when comparing classically defined final response criteria vs. data-driven response criteria. Although it is unclear as to why this discrepancy exists, possible explanations include a smaller sample size in the iTBS group, which could contribute to an increased artifact of variability in response to treatment. Additionally, it is possible that with reduced patient-technician contact time and reduced time spent in the potentially therapeutic environment of the clinic, that the iTBS group may have a more variable response to treatment. It is worth noting that in an accepted study using the same dataset, that no significant differences were found in the time in which patients responded to treatment or overall response rates between 10 Hz rTMS and iTBS on a variety of clinical outcomes (23).

Further corroborating existing literature that demonstrates a lack of differences in the clinical utility of 10 Hz rTMS and iTBS (15, 23), our current study did not find any significant differences between the two treatments in the predictive capabilities of early treatment improvement on final treatment response. Regarding the precision of the predictive capabilities, our data suggested that a 10% improvement cut-off by treatment 10 achieved the best NPV as a predictor of rTMS treatment response, whereas other published literature found 20% to have the highest NPV. One study showed a NPV of 72.3% when participants failed to reach 20% improvement at week two while using final outcomes of extended treatment courses of 10 Hz stimulation at the left DLPFC (12), and another which had ~80% NPV when using 1 Hz at the left DMPFC (13). Notably, our study focused on NPV as we felt this was the most important clinical information for rTMS practitioners to consider 10 treatments into an rTMS course.

### Strengths

Early treatment response has been demonstrated to be an effective clinical outcome prognosticator (24). Nonetheless, it is important to compare its clinical usefulness to biomarkers and their ability to predict treatment response. Interestingly, a study found that when examining potential predictive biomarkers such serum and plasma BDNF increases at week 1, as well as EEG markers, and comparing them to a 20% improvement criterion on MADRS scores at week two of SSRI treatment, clinical predictors were superior (25). This study found that the 2-week improvement evaluation had a 92% NPV, whereas the serologic studies had a NPV of 57%, and the EEG markers had a NPV of 72%–this further highlights the utility of early treatment response and negative predictive values in a clinical setting.

In general, our study found that non-response to iTBS or 10 Hz treatment for major depressive disorder can be predicted with 70 to 80% accuracy in patients exhibiting at least 10% improvement after 10 sessions. Our results will help inform future clinical trials designed to investigate what parameter changes may increase response rates at t_10_. In addition, although 70 to 80% accuracy may not be robust enough to create stringent treatment parameters for psychiatrists across the map, this data may help guide treatment decisions by identifying patients at risk for treatment non-response at the 2-week time point so therapeutic adjustments can be made to enhance treatment response. Some potential adaptations to existing treatment paradigms could include removing plasticity-impeding agents like benzodiazepines (26), accelerating TMS treatments with additional pulses (27), reducing stimulus intervals (28), increasing frequency (29), switching to bilateral stimulation (30), or other similar considerations.

### Limitations

Despite the benefit of naturalistic, observational study designs allowing a greater generalizability of results to other “real-world” populations, there are several limitations that impede interpretation of our results. One such limitation was that although patients received standard clinical TMS treatment, the non-randomized nature creates opportunities for several uncontrolled variables, such as comorbid psychiatric conditions or psychotropic medications to influence TMS response. This blurs our ability to comment on early treatment improvement to TMS treatment in isolation. In light of the lack of more stringent patient stratification, several studies exist that have already examined the efficacy of rTMS in the treatment of depression when evaluated against sham groups (3, 14, 31–33). Furthermore, to address these potential limiting factors, we advocate for additional multi-site trials to create larger participant pools so that subsequent studies may have the statistical power to control for some of the above confounders and further evaluate predictive capabilities of early treatment response in TMS. Another limitation worth noting is that studies using conditional-probability metrics such as negative predictive value have been previously critiqued for the use of seemingly inconsistent improvement thresholds (e.g., 0, 10, 20%) (34), which could create difficulties in comparing predictive capabilities in subsequent studies.

## Conclusion

To conclude, our naturalistic observational study, one of the first to directly compare the predictive capacity of early treatment improvement on ultimate treatment response between 10 Hz rTMS and iTBS, contributes to the growing consensus that there are no significant differences between the two modalities in the treatment outcomes for major depressive disorder. As the collection and analysis of biomarkers continues to remain expensive, time consuming, and inaccessible for many, studies like this further support the utility of easily attainable clinical predictors of treatment response in depression. TMS therapy often entails daily treatments for up to 6 weeks and beyond, requiring patients to take time off work or find transportation. The ability to forecast early in a treatment course a possible non-response to therapy will help both clinicians and patients decide if a parameter adjustment, or switch of therapy modalities entirely, may be warranted to maximize patient outcomes. Lastly, as iTBS sessions can be completed often ~30 min faster than 10 Hz rTMS, the lack of significant differences in prognostication of treatment response between the two modalities, as suggested here, may encourage future clinicians to increase preferential utilization of iTBS over 10 Hz rTMS to reduce the time burden on patients without sacrificing effectiveness.

## Data Availability Statement

The raw data supporting the conclusions of this article will be made available by the authors, upon reasonable request.

## Ethics Statement

The studies involving human participants were reviewed and approved by University of Iowa Institutional Review Board. The patients/participants provided their written informed consent to participate in this study. A waiver of consent was obtained for some subjects from the IRB if only clinical observational data was utilized.

## Author Contributions

NS: conceptualization, methodology, investigation, formal analysis, and writing-original draft. BP: methodology, data curation, resources, investigation, and reviewing and editing. PT: investigation, data curation, formal analysis, and reviewing and editing. NT: conceptualization, methodology, investigation, resources, supervision, reviewing and editing, and project administration. All authors contributed to the article and approved the submitted version.

## Funding

This study was supported in part by departmental funding from the Department of Psychiatry at the University of Iowa, Iowa City, IA; the INSPIRE Training Grant (5T32MH019113); the University of Iowa Clinical and Translational Science Award - NIH (UL1TR002537); the study was funded with support by the NIMH Career Development Award 1K23MH125145.

## Conflict of Interest

The authors declare that the research was conducted in the absence of any commercial or financial relationships that could be construed as a potential conflict of interest.

## Publisher's Note

All claims expressed in this article are solely those of the authors and do not necessarily represent those of their affiliated organizations, or those of the publisher, the editors and the reviewers. Any product that may be evaluated in this article, or claim that may be made by its manufacturer, is not guaranteed or endorsed by the publisher.
