# Corrigendum: Early improvement predicts clinical outcomes similarly in 10 Hz rTMS and iTBS therapy for depression

**DOI:** 10.3389/fpsyt.2024.1404381

**Published:** 2024-04-05

**Authors:** Nathen A. Spitz, Benjamin D. Pace, Patrick Ten Eyck, Nicholas T. Trapp

**Affiliations:** ^1^Department of Psychiatry, University of Iowa, Iowa City, IA, United States; ^2^Institute for Clinical and Translational Science, University of Iowa, Iowa City, IA, United States; ^3^Iowa Neuroscience Institute, University of Iowa, Iowa City, IA, United States

**Keywords:** depression, transcranial magnetic stimulation, theta-burst, clinical practice, observational study, prediction

In the published article, there was an error in **Table 2** as published. The data was incorrectly transposed into the table. The corrected **Table 2** and its caption appear below.

**Table 2:** Early improvement confusion matrices determining final treatment predictive capacity differences between 10 Hz rTMS and iTBS.

		10 Hz rTMS (n = 68)	iTBS (n = 37)	p value
Classically defined > 50% improvement				
**>20% improvement by treatment 10**				
**Sensitivity**		69.7	58.8	0.44
**Specificity**		80.0	65.0	0.22
**PPV**		76.7	58.8	0.20
**NPV**		73.7	65.0	0.49
**Total Accuracy**		75.0	62.2	0.17
**>10% improvement by treatment 10**				
**Sensitivity**		84.8	76.5	0.47
**Specificity**		62.9	50.0	0.35
**PPV**		68.3	56.5	0.35
**NPV**		81.5	71.4	0.46
**Total Accuracy**		73.5	62.2	0.23
**>0% improvement by treatment 10**				
**Sensitivity**		87.9	88.2	0.98
**Specificity**		31.4	15.0	0.18
**PPV**		54.7	46.9	0.48
**NPV**		73.3	60.0	0.58
**Total Accuracy**		58.8	48.6	0.32
KDE defined improvement (>40% 10 Hz, >45% iTBS)				
**>20% improvement by treatment 10**				
**Sensitivity**		67.6	61.1	0.64
**Specificity**		83.9	68.4	0.20
**PPV**		83.3	64.7	0.15
**NPV**		68.4	65.0	0.79
**Total Accuracy**		75.0	64.9	0.27
**>10% improvement by treatment 10**				
**Sensitivity**		83.8	77.8	0.59
**Specificity**		67.7	52.6	0.29
**PPV**		75.6	60.9	0.22
**NPV**		77.8	71.4	0.65
**Total Accuracy**		76.5	64.9	0.20
**>0% improvement by treatment 10**				
**Sensitivity**		86.5	88.9	0.80
**Specificity**		32.3	15.8	0.20
**PPV**		60.4	50.0	0.35
**NPV**		66.7	60.0	0.79
**Total Accuracy**		61.8	51.4	0.30

Using PHQ-9 score percent changes at treatment 10 and the final treatment, confusion matrices were calculated for 10Hz rTMS and iTBS across an array of improvement criteria. Classically defined improvement in scores is >50% from baseline. Kernel density estimate calculations were used to determine data-driven non-responder populations to create more stringent and improvement criteria, which was determined to be >40% for 10Hz rTMS and >45% for iTBS. rTMS, repetitive transcranial magnetic stimulation; iTBS, intermittent theta burst stimulation; PPV, positive predictive value; NPV, negative predictive value; KDE, kernel density estimate.

In the published article, there were text errors related to the mislabeled results in **Table 2** described above.

A correction has been made to the **Abstract** *Results*. This sentence previously stated:

“Results: For both modalities, the NPV related to degree of improvement at t10. NPV for 10 Hz was 80%, 63% and 46% at t10 in those who failed to improve >20, >10, and >0% respectively; while iTBS NPV rates were 65, 50, and 35%. There were not significant differences between protocols at any t10 cut-off assessed, whether research defined 50% improvement as response or data driven kernel density estimates (*p* = 0.22–0.44).”

The corrected sentence appears below:

“Results: For both modalities, the NPV related to degree of improvement at t10. NPV for 10 Hz was 74%, 82% and 73% at t10 in those who failed to improve >20, >10, and >0% respectively; while iTBS NPV rates were 65, 71, and 60%. There were not significant differences between protocols at any t10 cut-off assessed, whether research defined 50% improvement as response or data driven kernel density estimates (p = 0.46–0.79).”

A correction has been made to the **Abstract**, *Conclusion*. This sentence previously stated:

“Conclusion: Patients who fail to achieve >20% improvement by t10 with both 10Hz rTMS and iTBS therapies have ~70% chance of non-response to treatment.”

The corrected sentence appears below:

“Conclusion: Patients who fail to achieve >10% improvement by t10 with both 10Hz rTMS and iTBS therapies have 70-80% chance of non-response to treatment.”

A correction has been made to the **Results**, *Negative Predictive Analyses*, paragraph 1. This sentence previously stated:

“For participants who failed to reach >20% improvement at t10, the NPVs for 10Hz rTMS and iTBS were 80.0 and 65.0%, respectively: p = 0.22. When the improvement criterion was decreased to >10% improvement the NPV for 10Hz and iTBS decreased to 62.9 and 50.0%: p = 0.35. Lastly, at >0% improvement the NPV for 10Hz and iTBS decreased further to 45.7 and 5.0%: p = 0.44.”

The corrected sentence appears below:

“For participants who failed to reach >20% improvement at t10, the NPVs for 10Hz rTMS and iTBS were 73.7 and 65.0%, respectively: p = 0.49. When the improvement criterion was decreased to >10% improvement the NPVs for 10Hz and iTBS were 81.5 and 71.4%: p = 0.46. Lastly, at >0% improvement the NPVs for 10Hz and iTBS decreased to 73.3 and 60.0%: p = 0.58.”

A correction has been made to the **Results**, *Negative Predictive Analyses*, paragraph 2. The sentence previously stated:

“At >20% improvement at t10, the NPV for 10Hz rTMS and iTBS were 83.9 and 68.4%, respectively: p =0.20. Then at >10% improvement the NPV for 10Hz and iTBS decreased to 67.7 and 52.6%: p = 0.28. Lastly, at >0% improvement the NPV for 10Hz and iTBS decreased further to 48.4 and 36.8%: p = 0.44.”

The corrected sentence appears below:

“At >20% improvement at t10, the NPVs for 10Hz rTMS and iTBS were 68.4 and 65.0%, respectively: p =0.79. Then at >10% improvement the NPVs for 10Hz and iTBS were 77.8 and 71.4%: p = 0.65. Lastly, at >0% improvement the NPVs for 10Hz and iTBS decreased to 66.7 and 60.0%: p = 0.79.”

A correction has been made to the **Discussion**, paragraph 1. This sentence previously stated:

“Our data demonstrated that as the early treatment improvement criterion increased, so did the NPVs of both 10Hz rTMS and iTBS, while maintaining no significant differences between the two modalities.”

The corrected sentence appears below:

“Our data demonstrated no significant differences between the two modalities.”

A correction has been made in the **Discussion**, paragraph 3. This sentence previously stated:

“Regarding the precision of the predictive capabilities, our data was comparable with previous studies in that a 20% improvement cut-off by treatment 10 achieved the best NPV as a predictor of rTMS treatment response.”

The corrected sentence appears below:

“Regarding the precision of the predictive capabilities, our data suggested that a 10% improvement cut-off by treatment 10 achieved the best NPV as a predictor of rTMS treatment response, whereas other published literature found 20% to have the highest NPV.”

A correction has been made to the **Discussion**, Strengths, paragraph 2. This sentence previously stated:

“In general, our study found that non-response to iTBS or 10Hz treatment for major depressive disorder can be predicted with ~70% accuracy in patients exhibiting at least 20% improvement after 10 sessions. Our results will help inform future clinical trials designed to investigate what parameter changes may increase response rates at t10. In addition, although ~70% accuracy may not be robust enough to create stringent treatment parameters for psychiatrists across the map, this data may help guide treatment decisions by identifying patients at risk for treatment non-response at the 2-week time point so therapeutic adjustments can be made to enhance treatment response.”

The corrected sentence appears below:

“In general, our study found that non-response to iTBS or 10 Hz treatment for major depressive disorder can be predicted with 70 to 80% accuracy in patients exhibiting at least 10% improvement after 10 sessions. Our results will help inform future clinical trials designed to investigate what parameter changes may increase response rates at t10. In addition, although 70 to 80% accuracy may not be robust enough to create stringent treatment parameters for psychiatrists across the map, this data may help guide treatment decisions by identifying patients at risk for treatment non-response at the 2-week time point so therapeutic adjustments can be made to enhance treatment response.”

The authors apologize for these errors and state that these do not change the scientific conclusions of the article in any way. The original article has been updated.

